# Characterization of a Hepatitis Outbreak in Children, 2021 to 2022

**DOI:** 10.1001/jamanetworkopen.2022.37091

**Published:** 2022-10-18

**Authors:** Emma C. Alexander, Akash Deep

**Affiliations:** 1 Paediatric Intensive Care Unit, King’s College Hospital NHS (National Health Service) Foundation Trust, London, United Kingdom

## Abstract

**Importance:**

After a cluster of pediatric cases of hepatitis of unknown etiology were identified in Scotland in March 2022, the World Health Organization published an outbreak alert, and more than 1010 probable cases were reported. Some cases progressed to acute liver failure and required liver transplant. Although many patients had positive results for adenovirus on polymerase chain reaction testing from whole blood samples and/or reported recent COVID-19 infection (with or without seropositivity), the precise pathogenesis remains unclear despite the high potential morbidity of this condition.

**Objective:**

To summarize the currently available evidence regarding novel pediatric hepatitis of unknown etiology (or novel hepatitis), encompassing case numbers, testing, management, and outcomes.

**Evidence Review:**

A rapid review of the literature from April 1, 2021, to August 30, 2022, aimed to identify all available published case series and case-control studies of novel hepatitis. The search included PubMed and references and citations of short-listed studies.

**Findings:**

A total of 22 available case series and case-control studies describing 1643 cases were identified, with 120 children (7.3%) receiving liver transplants and 24 deaths (1.5%). Outcome reporting and testing for adenovirus and SARS-CoV-2 was incomplete. Assessment of disease severity and management was mixed and results regarding testing for adenovirus and SARS-CoV-2 were inconsistent for both serological testing and testing of explant or biopsy liver samples. More recent studies suggest a more plausible role for adenovirus and/or adeno-associated virus 2.

**Conclusions and Relevance:**

This systematic review without meta-analysis describes the challenge posed by hepatitis of unknown etiology in terms of investigation and management, with many cases progressing to acute liver failure. The lack of clarity regarding pathogenesis means that these children may be missing the potential for targeted therapies to improve outcomes and avert the need for transplant. Clinicians, immunologists, and epidemiologists must collaborate to investigate the pathogenesis of this novel hepatitis.

## Key Points

**Question:**

What is the available evidence regarding cases of hepatitis of unknown etiology in children in 2021 to 2022?

**Findings:**

This systematic review without meta-analysis identified 1643 cases across 22 studies, with 120 children (7%) receiving liver transplants and 24 deaths (1%). Testing focused on adenovirus, SARS-CoV-2, and adeno-associated virus 2.

**Meaning:**

These findings suggest that the cause of novel hepatitis in children remains undetermined, but the potential role of adenovirus, adeno-associated virus 2, and SARS-CoV-2 requires further exploration in multicenter collaborative studies.

## Teaser

This narrative review investigates the current published case series and case-control studies of pediatric patients with acute hepatitis of unknown etiology.

## Introduction

After an unexpected cluster of pediatric cases of hepatitis of unknown etiology was identified in Scotland in March 2022,^1^ the World Health Organization (WHO) announced an outbreak alert. Since then, a review covering October 1, 2021, to July 18, 2022, identified at least 1010 probable cases of acute hepatitis of unknown etiology from 35 countries, including 334 from the US and 272 from the UK.^2^ Among these probable cases, many of the individuals have experienced progression to acute liver failure (ALF), 46 (4.6%) have required liver transplants, and 22 (2.2%) have died.

Hepatitis refers to inflammation of the liver. Presenting symptoms include vomiting, abdominal pain, jaundice, weakness, and lethargy. Hepatitis is commonly caused by viruses, such as hepatitis A through E viruses, as well as herpes simplex, enterovirus, Epstein-Barr virus, and cytomegalovirus.^3^ However, for many patients, no cause is ever found. These cases are alternately termed *non–A to E*, *seronegative*, or *indeterminate hepatitis*.^4^ Most children who develop indeterminate or non–A to E hepatitis recover alone with supportive management. However, some cases lead to complications, including ALF or chronic liver disease. In developed countries, approximately 40% of cases of ALF are identified as indeterminate.^5^ Patients who develop indeterminate ALF have a worse prognosis and are less likely to achieve native liver survival than those who develop ALF due to specific causes such as acetaminophen (paracetamol) toxicity.^4,6^ By definition, the cause of indeterminate ALF is unknown. Infiltration of cytotoxic CD8^+^ T cells with increased clonality has been demonstrated in patients with indeterminate pediatric ALF compared with cases of pediatric ALF with known causes, which suggests antigens, and hence possibly an undetected infection, as a driver of T-cell expansion, rather than a nonspecific inflammatory response.^7,8^ Other possible causes include an unrecognized autoimmune disease, unidentified drug toxicity or drug reaction, and occult metabolic or genetic disorder.^4^ Thus, a thorough diagnostic workup is necessary for all children presenting with hepatitis, to enable initiation of etiology-specific therapies.^9^

Since the novel hepatitis outbreak was identified, the WHO has published monthly updates regarding case numbers and regions affected, including information available on testing for adenovirus and SARS-CoV-2. The definition of a probable case used by WHO is that of a “person presenting with an acute hepatitis (non A-E) with serum transaminase >500 IU/L (AST [aspartate aminotransferase] or ALT [alanine aminotransferase]), who is 16 years and younger, since 1 October 2021)”.^2^ Cases with a known alternative explanation for their presentation are excluded. Cases are predominantly children younger than 6 years.^2^

There has been much speculation on the underlying mechanism that has led to clustering of this condition, and why some cases progress to ALF and require liver transplant. Many patients had positive results for adenovirus on polymerase chain reaction (PCR) testing of whole blood samples and/or reported recent COVID-19 infection, but the precise pathogenesis has remained unclear. This report presents the results of a rapid review of the currently published case series and case-control studies of this condition.

## Methods

We conducted a systematic review without meta-analysis from April 1, 2021, to August 30, 2022, to identify all available published case series and case-control studies of novel hepatitis. Titles and abstracts were searched in PubMed for the terms *pediatric* or *children* and *hepatitis* to August 30, 2022, to identify the relevant studies. The full texts of relevant titles and abstracts were then reviewed to determine inclusion. Citations and references of included studies were also searched to identify any additional studies, which identified some non–peer-reviewed studies. Both authors verified inclusion of the relevant studies. In line with the Checklist for Reporting Case Series, we have described the study hypotheses in the Table and eligibility criteria for each study, treatments administered, comparisons, and statistical methods if applicable in the eTable in the Supplement.

**Table.** Hypotheses Regarding the Etiology of Novel Hepatitis

Source	Country	Hypothesis
Marsh et al,^1^ 2022	Scotland	“At the time of publication, the leading hypotheses centre around adenovirus—either a new variant with a distinct clinical syndrome or a routinely circulating variant that is more severely impacting younger children who are immunologically naive.”
Deep et al,^10^ 2022	London, UK	“The presence of adenovirus in these children raises a hypothesis of SARS-CoV-2 superantigen-mediated disease potentiated by a second virus.”
UK Health Security Agency,^11^ 2022	UK	“The association between adenovirus infection and cases is helpful…. The lack of apparent direct toxic effects of the virus on liver tissue and other results suggests this is part of a multiple-step process.”
Cooper et al,^12^ 2022	Israel	“Our hypothesis that the mechanism of liver manifestation is either a postinfectious immune reaction similar to MIS-C, or an immune dysregulation causing priming to other infectious agent such as adenovirus by a prior infection with SARS-CoV-2, is supported by our histology findings.”
de Kleine et al,^13^ 2022	ERN RARE-LIVER survey of 22 European countries and Israel	“We did not detect adenoviruses in the majority of patients, nor another uniform viral infection…. No increase in virus-derived hepatitis could be detected at this point in time within the whole group of participating centres.”
CDC Health Alert Network,^14^ 2022	US	“A possible association between pediatric hepatitis and adenovirus infection is under investigation.”
Baker et al,^15,16^ 2022; Gutierrez Sanchez et al,^17^ 2022	Alabama, US	“This cluster, along with recently identified possible cases in Europe, suggests that adenovirus should be considered in the differential diagnosis of acute hepatitis of unknown etiology among children.” and “…Whether human adenovirus was causative remains unclear. Sequencing results suggest that if human adenovirus was causative, this was not an outbreak driven by a single strain.”
Joint ECDC–WHO Regional Office for Europe,^18^ 2022	EU/EEA	“The current leading hypothesis is that a cofactor affecting young children having an adenovirus infection, which would be mild in normal circumstances, triggers a more severe infection or immune-mediated liver damage.”
Antala et al,^19^ 2022	US	“SARS-CoV-2 infection should be considered as the cause of acute severe hepatitis even in patients without significant respiratory or systemic symptoms.”
Ratho et al,^20^ 2021 (preprint)	India	“With the emergence of newer variants of concern including the Delta variant which predominated the second wave of infections in India and has now spread to more than 142 countries with changing presentations, CAH-C might be one of them.”
van Beek et al,^21^ 2022	Survey of hospitals in 17 European and 7 non-European countries	“Adenovirus F type 41 was detected in 18 cases with available typing data (91/126 cases [72%] tested positive for adenovirus), and has been suggested as a causative agent with or without a cofactor.”
Israel Ministry of Health,^22^ 2022; Efrati,^23^ 2022	Israel	“These reports are currently reviewed.”
Kambhampati et al,^24^ 2022	US	“The potential role of adenovirus in the etiology of the newly reported hepatitis cases is unknown; ongoing investigations are assessing this hypothesis along with the possible role of other factors, including current or past infections with SARS-CoV-2.”
Einberg and Fischler,^25^ 2022	Sweden	“So far, adenovirus infection with type 41 seems to be of etiological importance, but the importance of other virological, immunological or toxicological factors remains to be investigated.”
Wollants et al,^26^ 2022 (preprint)	Belgium	“If either or both [of adenovirus or SARS-CoV-2 infections] do however contribute, only a small minority of infections are likely to lead to severe hepatitis.”
Lexmond et al,^27^ 2022	the Netherlands	“While these studies are underway, the relationship between acute severe hepatitis in children and our cluster of indeterminate PALF patients remains speculative.”
Di Dato et al,^28^ 2022	Italy	“The results from our study suggest a poor role of Sars-CoV-2 and adenovirus infection in determining severe acute hepatitis.”
Kelgeri et al,^29^ 2022	Birmingham, UK	“…Human adenovirus was isolated in most of the children, but its role in the pathogenesis of this illness has not been established.”
Romaní Vidal et al,^30^ 2022	20 countries in WHO European region	“Aetiological studies are needed to ascertain if adenovirus plays a role in this possible emergence of hepatitis cases in children and, if confirmed, the mechanisms that could be involved.”
Ho et al,^31^ 2022 (preprint)	Scotland	“Acute non–A-E paediatric hepatitis is associated with the presence of AAV2 infection, which could represent a primary pathogen or a useful biomarker of recent HAdV or HHV6B infection.”
Morfopoulou et al,^32^ 2022 (preprint)	UK	“The potential that AAV2, although not previously associated with disease, may, together with HAdV-F41 and/or HHV-6, be causally implicated in the outbreak of unexplained hepatitis, requires further investigation.”
Cates et al,^33^ 2022	US	“Current US data do not suggest an increase in pediatric hepatitis of unknown etiology or percent positivity in adenovirus types 40/41 over baseline levels. Additional hypotheses are under investigation, including the potential role of previous SARS-CoV-2 infection and adeno-associated virus-2….”

Abbreviations: AAV2, adeno-associated virus 2; CAH-C, COVID-19–associated hepatitis in children; ECDC, European Centre for Disease Prevention and Control; ERN RARE-LIVER, European Reference Network on Hepatological Diseases; EU/EEA, European Union/European Economic Area; HAdV, human adenovirus; HHV6B, human herpesvirus 6B; MIS-C, multisystem inflammatory syndrome in children; PALF, pediatric acute liver failure; WHO, World Health Organization.

## Results

We identified a total of 22 studies that included 1643 cases. We have summarized the available studies for 22 distinct populations in the eTable in the Supplement, including country, age, case definition, testing for adenovirus and/or SARS-CoV-2, treatments, and outcomes.^1,10,11,12,13,14,15,16,17,18,19,20,21,22,23,24,25,26,27,28,29,30,31,32,33^ The Table summarizes the hypotheses raised by these case series. Among the 1643 cases, 120 children (7.3%) received liver transplants and 24 (1.5%) died. Some patients may have appeared across multiple studies and some outcomes were not reported. Most studies used the WHO case definition.^2^ Countries included the UK,^1,10,11,29,31,32^ Israel,^12,22,23^ the US,^14,15,16,17,19,24,33^ India,^20^ Sweden,^25^ Belgium,^26^ the Netherlands,^27^ Italy,^28^ and several multicountry collaboratives.^13,18,21,30^

Among peer-reviewed series, 9 children from Alabama are described who presented with hepatitis of unknown etiology and positive test findings for adenovirus on whole blood testing between October 2021 and February 2022.^15,16,17^ All 9 children had PCR test results negative for SARS-CoV-2; antibody testing was not performed. Seven children survived with native liver, and 2 survived after transplant. Initial immunohistochemical and electron microscopy examination of biopsy samples showed no evidence of adenovirus or SARS-CoV-2. However, PCR testing for adenovirus later yielded positive findings on 3 of the 6 samples.^17^

In the UK, our center at King’s College Hospital, London, published a series including 8 children admitted to the pediatric intensive care unit with ALF and multiple systems organ failure (MSOF).^10^ Most of these patients required neuromonitoring, neuroprotection, and extracorporeal therapies. Although results of blood virology revealed adenovirus DNA, all patients undergoing transplant had negative findings for adenovirus on explant histopathologic evaluation. All 8 patients had PCR test results negative for SARS-CoV-2, although 75% had positive findings for antibodies on serological testing. All 8 children survived, but 6 (75%) required liver transplant.

In Birmingham, UK, a series of 44 children with hepatitis younger than 10 years was reported, of whom 6 required transplant and all 44 survived.^29^ Twenty-five of 27 (93%) patients undergoing testing for adenovirus from whole blood samples had positive findings, compared with a positivity rate of 28% for SARS-CoV-2 on molecular test results and 38% on serological test results. As in the London series, there were no viral inclusions, and the results on immunohistochemical testing of liver samples were negative. However, of 3 explant samples that were tested, adenovirus was identified on PCR assays, with sequencing showing similarity to adenovirus type 41F.

A series from Israel described 5 children, all with test findings positive for SARS-CoV-2 in the recent past.^12^ Three of these children developed hepatitis with cholestasis, and 2 experienced rapid progression to ALF and required transplant. The former cohort was treated with corticosteroids, as for a postinfectious inflammatory disorder, and all 5 children survived. Likewise, all had negative immunostaining findings for adenovirus or SARS-CoV-2 on explant or liver biopsy specimens.

A peer-reviewed publication of cases submitted to the European Surveillance System summarized 427 cases reported by 20 countries.^30^ Overall, 174 of 325 participants (53.5%) undergoing testing had positive findings for adenovirus, 29 of 280 (10.4%) had positive PCR findings for SARS-CoV-2, and 31 of 47 (66.0%) had positive serological test findings for SARS-CoV-2. For those with information regarding outcomes, 84 of 261 (32.2%) were admitted to the intensive care unit or the hemodialysis unit, and 18 of 208 (8.7%) received a transplant. Other series, including preprints^20,26,31,32^ and technical reports,^11,14,18,22,23^ are summarized in the eTable in the Supplement.

## Discussion

### Pathophysiology, Including the Role of Adenovirus and SARS-CoV-2

A great deal of uncertainty and speculation remains regarding the pathogenesis of novel hepatitis. The role of adenovirus as a causative factor for at least some cases was theorized early and remains relevant. The initial cases identified in the UK had a perceived high positivity rate for adenovirus (with 170 of 258 [65.9%] cases having positive test results as of July 4, 2022),^11^ most commonly adenovirus type 41F. This rate exceeds the normal population prevalence of adenovirus in the UK, which during the past year has reached approximately 13% positivity at peak periods among children aged 0 to 4 years.^34^ From a UK matched case-control study,^11^ the adjusted odds ratio of having concomitant adenovirus in cases of acute hepatitis of unknown etiology compared with healthy controls was 35.27 (95% CI, 15.23-81.68). Of note, optimal detection of adenovirus was from whole blood samples, rather than stool or nasopharyngeal samples, which were used in many series.^11^ Adenovirus type 41F has previously been associated with gastrointestinal tract illness, but hepatitis and ALF are rare consequences.^35^ Adenovirus overall is a recognized, although very rare, cause of ALF in patients with immunocompetence.^36^

SARS-CoV-2 has also been implicated, acutely and after serological testing. In the latest WHO report published on July 12, 2022,^2^ of 1010 probable cases, 78 (7.7%) had PCR test findings positive for SARS-CoV-2, compared with 209 (20.7%) PCR test findings positive for adenovirus, although not all cases were tested for either virus. Detailed results from serological testing for SARS-CoV-2 are at present limited. Positive results cannot differentiate natural from vaccine-induced exposure and cannot determine the time frame of exposure. Population prevalence of SARS-CoV-2 varied significantly by country during this time. A matched case-control study in the UK did not identify a difference in positivity for SARS-CoV-2 in cases compared with controls.^11^ However, hepatitis in adults is not uncommon in patients with SARS-CoV-2. In a retrospective cohort study of 2273 adult patients,^37^ published in May 2020, 45% developed a mild liver injury and 6.4% developed severe liver injury. A case report was published in August 2021 of ALF in a previously healthy female adolescent,^38^ with a biopsy result showing replicating SARS-CoV-2 RNA. Several case series listed in the eTable in the Supplement implicated SARS-CoV-2.^12,19,20^

A proposed unifying theory regarding the role of SARS-CoV-2 relates to superantigens.^39^ A sequence motif on the SARS-CoV-2 spike protein has been identified as being similar in sequence and structure to the known superantigen staphylococcal enterotoxin B.^40^ Thus, the SARS-CoV-2 spike protein may be able to elicit broad nonspecific T-cell activation in a similar manner. In addition, previous immunological studies in mice have shown that systemic adenovirus infection significantly increased the severity of liver injury induced by staphylococcal enterotoxin B.^41^ It has been demonstrated that in multisystem inflammatory syndrome in children, prolonged presence of SARS-CoV-2 in the intestinal tract is associated with increased circulating zonulin levels and subsequent increased intestinal permeability and antigenemia.^42^ Perhaps, in certain susceptible children harboring gut reservoirs of SARS-CoV-2, who are then sensitized by adenovirus 41F or adeno-associated virus 2 (AAV-2) (which will be discussed below), gut leakage and subsequent viremia could trigger a cytokine storm and subsequent hepatitis and ALF in patients with severe disease.

The difficulty with implicating either adenovirus or SARS-CoV-2 relates to the observation that cases are not consistently positive for one or the other, and early reports of testing of explant or biopsy samples reportedly had negative results for both these viruses.^10,12,15^ The first conclusive reports of adenovirus in liver samples occurred in July 2022, after 2 publications in the *New England Journal of Medicine*, from Birmingham, UK, and Alabama^17,29^ reported positive results for 3 patients each after PCR testing of liver tissue. The UK Health Security Agency^11^ suggested that it is possible adenovirus is part of a multistep process, and other hypotheses are also possible, including environmental toxins, other viruses, and genetic factors. Following this line of investigation, among other viruses, a number of studies have reported the presence of AAV-2,^11,13,25^ which is a dependoparvovirus that usually requires coinfection with another virus to successfully replicate.

In July 2022, 2 preprints (summarized in the eTable in the Supplement)^31,32^ from the UK described the potentially significant role of AAV-2. In 1 case-control study from Scotland,^31^ AAV-2 was detected in 9 of 9 plasma samples and 4 of 4 liver biopsy samples from 9 cases, alongside coinfection with adenovirus or human herpesvirus 6B for most cases. Eight of the 9 cases carried the HLA-DRB1*04:01 gene variant, suggesting a possible variance in immune response being related to novel hepatitis. A second study of 28 cases and 136 controls^32^ reported all 5 explanted livers and 10 of 11 blood samples from nontransplant cases were positive for AAV-2. All liver samples and 21 of 23 other cases also had PCR results positive for adenovirus (the liver samples suggested lower viral titres for adenovirus than AAV-2).^32^ The HLA-DRB1*04:01 variant was found in 4 of 5 cases receiving a transplant in the series. In both studies, prevalence of AAV-2 and/or adenovirus was significantly lower in controls, among whom almost none had positive results. Viral proteins and/or particles were not found in the explanted livers, suggesting that an immune response, rather than lytic injury, is key. Consequently, on July 27, an editorial published in *The Lancet Child & Adolescent Health* suggested that based on the available evidence, coinfection with adenovirus and AAV-2 is at present the most probable causal mechanism.^43^ Genetically predisposed individuals may be at heightened risk of a dysfunctional immune response, and hence liver injury.

### Management and Treatment Offered

In general, management of indeterminate hepatitis is supportive, and most patients recover spontaneously. However, those with progression to ALF have a high level of morbidity, with 20% requiring liver transplant,^44,45^ and a mortality of approximately 5% to 10% among all patients, which is greater in those who develop indeterminate ALF.^46^ Most deaths are secondary to cerebral edema, sepsis, and MSOF. As a result, high-quality multisystem monitoring is vital, in particular neuromonitoring to identify early signs of encephalopathy and cerebral edema and to take neuroprotective measures.^47^

In our review of available case series of novel hepatitis, many children experienced progression to ALF and 17 of 20 series with available outcomes included some patients who required liver transplant. Some children were critically ill, requiring multiorgan support; in the London series 7 of 8 required noradrenaline, and 5 of 8 were treated with kidney replacement therapy.^10^ All 5 patients in the Netherlands series developed at least grade I encephalopathy.^27^ These series are naturally selective with predominance of reporting on the most unwell cases. However, the potential for these children to experience deterioration should be recognized.

Regarding therapies, a number of studies reported use of corticosteroids, including studies from Israel,^12^ India,^20^ Scotland,^31^ and the US.^15,17,19^ Children with a disease process propagated by a dysfunctional immune response may be thought likely to respond to immunosuppression. However, in the presence of adenoviremia for some patients, many clinicians might hesitate before prescribing corticosteroids. Until pathogenesis is clarified, it would also be difficult for any clinician to await response to immunosuppression rather than proceeding to transplant, in a child with MSOF at imminent risk of death and satisfying the liver transplant listing criteria.

Among other therapies, cidofovir, a selective inhibitor of viral DNA polymerases, has previously been used to treat patients with severe adenovirus infection, even in the absence of immune dysfunction.^48^ Cidofovir was used in 2 centers in the UK^10,29^ and the US.^15,17^ Cidofovir is used in these cases because patients with ALF, MSOF, and adenoviremia are inherently in a state of innate immune dysfunction.^49^

### Is There a True Increase in Prevalence?

One ongoing challenging question is whether there is in fact a true increase in prevalence of non–A to E hepatitis, or whether the current interest reflects heightened alertness in the context of the COVID-19 pandemic. Several studies have attempted to quantify this, with mixed results.^11,13,21,24,28^ The European Reference Network on Hepatological Diseases^13^ conducted a survey of its member centers and, across all 34 participating centers, 22 reported no increase in children with severe hepatitis during the relevant time period, and 10 reported that no cases of pediatric hepatitis were treated at all. In a smaller survey of 27 Italian pediatric centers between January and May 2022, no center reported that cases of severe acute hepatitis had increased compared with the preceding 3 years.^28^ In the US, data related to hepatitis and adenovirus in children from 4 large administrative databases were used to compare data from October 2021 through March 2022 with prepandemic baseline data.^24^ There was no increase in coding for hepatitis-associated emergency department attendances and hospitalizations in children aged 0 to 11 years, nor was there a significant increase in the number of monthly liver transplants. In the UK, coding for emergency admissions in children aged 1 to 4 years with liver-related illnesses and jaundice both showed a large increase.^11^ The largest survey^21^ was a collaboration between various European research networks and received responses from 52 hospitals in 17 European and 7 non-European countries. Probable cases matching the case definition were elevated in 6 of 20 hospitals with relevant available data (in the UK, Italy, Spain, Sweden, Ukraine, and Israel).

It appears that across most countries, there remains insufficient evidence of a significant increase in case numbers of novel hepatitis. There are some notable exceptions, including in the UK, where case numbers are 10 times as high as in usual years.^50^ It is unknown why this may be the case. An interesting analysis compared the number of cases of unexplained severe hepatitis in children across 38 Organisation for Economic Cooperation and Development member countries plus Romania with the numbers of confirmed Omicron (B.1.1.529) variant cases and found that countries reporting focal hepatitis cases experienced higher population burdens of Omicron relative to those without any focal hepatitis cases.^51^ Another theory is that levels of baseline immunity to adenovirus and/or AAV-2, made lower by factors such as good sanitation, stringent lockdowns, or other factors varying between countries, could increase predisposition to viral infection and broad immunological responses leading to liver damage.^52^ However, many potential factors remain. High-quality quantitative analyses with a greater pool of case numbers across a longer period, with more information on testing, management, and outcomes, could clarify this question. The European Society of Pediatric Gastroenterology, Hepatology and Nutrition^53^ in July published a research agenda for this condition to guide subsequent research efforts.

### Limitations

Limitations of this study include a search that was limited to 1 database. We aimed to mitigate this by searching the citations of all included studies, although we may have missed some small series and nonindexed work. Some studies were not peer reviewed, and some patients may have been included in multiple studies. All included series were in the English language. Last, owing to study design, conclusions about pathophysiology cannot be decisive.

## Conclusions

The findings of this review suggest that clinicians, immunologists, and epidemiologists must collaborate to investigate the epidemiology and pathogenesis of this novel form of hepatitis. It will be challenging, although necessary, to determine the role of adenovirus, AAV-2, and particularly SARS-CoV-2 in the current context, given high baseline levels of infection and seropositivity for the latter. Understanding the pathophysiology for the current outbreak can increase our readiness to treat the next outbreak and offer appropriate treatments. If a mechanism related to a postinfectious inflammatory process is found, the use of corticosteroids or immunomodulators at an early stage may prevent these children from experiencing progression to ALF and allow them to avoid liver transplant.

## Notes

Supplement. **eTable.** Case Series/Case Control Studies of Severe Hepatitis/Acute Liver Failure in Children, 2021 to 2022
eReferences
