# Supplementary material for: Characterization of a Hepatitis Outbreak in Children, 2021 to 2022
Source: JAMA Netw Open. 2022 Oct 18;5(10):e2237091. doi: 10.1001/jamanetworkopen.2022.37091 (PMC9579900; doi:10.1001/jamanetworkopen.2022.37091)
Supplement: Supplement. — eTable. Case Series/Case Control Studies of Severe Hepatitis/Acute Liver Failure in Children, 2021 to 2022 eReferences [file jamanetwopen-e2237091-s001.pdf]

Supplementary Online Content

Alexander EC, Deep A. Characterization of a hepatitis outbreak in children, 2021 to 2022. *JAMA Netw Open*. 2022;5(10):e2237091. doi:10.1001/jamanetworkopen.2022.37091

eTable. Case Series/Case Control Studies of Severe Hepatitis/Acute Liver Failure in Children, 2021 to 2022
eReferences

This supplementary material has been provided by the authors to give readers additional information about their work.

© 2022 Alexander EC et al. *JAMA Network Open*.

eTable. Case Series/Case Control Studies of Severe Hepatitis/Acute Liver Failure in Children, 2021 to 2022

	Location	Sample Size	Time	Age	Ethnicity	Case definition	Treatments offered	Survival	Transplant Outcomes	PCR for SARS-CoV-2 positive?	SARS-CoV-2 antibody positive	Adenovirus testing	Liver analyses
**Deep et al. (2022)[1]**	London, United Kingdom	n=8	February-May 2022	Median 2 years 9 months	100% white (unspecified)	Acute Liver Failure due to hepatitis	5/8 (62.5%) treated with Kidney Replacement Therapy, 7 (87.5%) required noradrenaline, all patients positive for adenovirus received cidofovir, all received N-acetylcysteine.	100% survival	25% survival with native liver (SNL), 75% survived with liver transplant (SLT)	100% No	75% Yes, 12.5% No, 12.5% Indeterminate	100% Adenovirus DNA positive from blood (87.5% at admission, 12.5% after admission)	100% (n=6) adenovirus immunostaining negative on explant histopathology.

© 2022 Alexander EC et al. *JAMA Network Open*.

**UK Health Security Agency Technical briefing 4[2]**	United Kingdom / England	n=195	January 2022 – 4 July 2022	From cases in England, median age 3 years (IQR 2-5).	In England majority white ethnicity (163 of 195, 83.6%)	Acute non-A-E hepatitis with serum transaminases greater than 500 IU/l	Not reported.	100% survival	n=15 (5.5%) Transplanted	In UK, 36 of 237 (15.2%) of cases were PCR positive around admission. No significant difference in PCR positivity when comparing cases to community controls.	No significant difference in antibody positivity when comparing hepatitis cases to NHS patient controls.	170 of 258 tested subjects (65.9%) were positive for adenovirus.	Of 5 explanted livers analysed by GOSH, high levels of AAV2 were detected, along with low levels of adenovirus and human herpesvirus 6B. However viral particle or protein were not detected, suggesting a lack of lytic infection. In Glasgow, 4 of 4 cases had AAV2 isolated in liver specimens. SARS-CoV-2 was not detected in any liver samples.
**Cooper et al. (2022)[3]**	Israel	n=5	2021	1x 3 months old, 1x 5 months old, 2 x 8 years old, 1x 13 years old	Not reported.	Liver involvement after COVID-19 infection. n=2 ALF, n=3 acute hepatitis with cholestasis	The three children with hepatitis and cholestasis were treated with systemic steroids.	100% survival	n=2 with ALF both transplanted	n=1 SARS-CoV-2 positive 21 days pre-ALF, n=1 not reported, n=1 SARS-CoV-2 positive 130 days pre-hepatitis, n=1 PCR positive 94 days pre-hepatitis, n=1 PCR positive; developed transient hepatitis then ALF 53 days later	In 1 case where acute test not reported, SARS-CoV-2 IgG was positive.	Three patients tested for adenovirus PCR from whole blood, positive for one.	100% negative on immunostaining for adenovirus or SARS-CoV-2 on explant or liver biopsy.

© 2022 Alexander EC et al. *JAMA Network Open*.

**de Kleine et al. (2022)[4]**	ERN RARE-LIVER survey of 22 European countries and Israel	n=64	1 January – 26 April 2022	Mean 7.7 years, range 28 days – 16 years	Not reported.	Severe hepatitis or paediatric acute liver failure	Not reported.	6.7% (n=4) died, 3 pre-transplant, 1 post-transplant	6.7% transplanted (n=4)	5% (n=3) had active SARS-CoV-2 infection at admission. Four patients with SARS-CoV-2 (6.3%) had a possible cause for presentation.	Not reported.	Adenovirus was positive for four children.	Not reported.
**Marsh et al. (2022)[5]**	Scotland	n=13	1 January – 12 April 2022	Median 3.9 years (IQR 3.6-4.6)	All white Scottish.	Severe hepatitis of unknown aetiology.	Not reported.	100% survival	7.7% (n=1) transplanted	5 of 13 had a recent positive test.	Not reported.	5 of 11 with results available were adenovirus positive by PCR.	Not reported.
**CDC Health Alert Network[6]**	United States	n=109	Up to May 5 2022	Not reported.	Not reported.	Hepatitis of unknown aetiology	Not reported.	4.6% (n=5) deaths	14% transplanted	Not reported.	Not reported.	"More than half" have tested positive for adenovirus.	Not reported.

© 2022 Alexander EC et al. *JAMA Network Open*.

**Baker et al. (2022)[7,8] and Gutierrez Sanchez et al. (2022)[9]**	Alabama, United States	n=9	October 2021-February 2022	Median age 2 years 11 months	9 White; 6 Hispanic, 3 non-Hispanic	ICD-10 definition of hepatitis in children <18 years admitted with acute hepatitis who also tested PCR positive for adenovirus	Two of three patients who developed acute liver failure were treated with cidofovir and steroids and transplanted. Both transplanted children received extracorporeal liver support.	100% survival	22.2% (2/9) transplanted. Adenovirus viraemia levels were higher in those who were transplanted.	All patients received nucleic acid amplification testing for SARS-CoV-2, all negative.	No patient had a documented history of previous SARS-CoV-2 infection, no formal antibody testing occurred.	100% (n=9) of patients were positive for adenovirus from whole blood PCR testing	Liver biopsies from six patients showed no viral inclusions or immunohistochemical evidence of adenovirus or viral particles on electron microscopy. However, three of six patients were later reported as PCR positive for adenovirus on formalin fixed paraffin embedded or fresh frozen liver tissue. FFPE biopsy specimens from all six were negative for SARS-CoV-2.
**Joint European Centre for Disease Prevention and Control-WHO regional office[10]**	EU/EEA	n=205 (excluding UK)	1 January 2022 – 30 June 2022	Mode 3 years. 76.1% 5 or younger.	Not reported.	Hepatitis of unknown origin in children aged 16 years or below (using WHO definition)	Not reported.	Of 302 cases with outcome available, one death.	Of 242 cases with available information, 20 (8.3%) transplanted.	10.9% (35 of 322) PCR positive for SARS-CoV-2.	63.9% (39 of 61) serology positive.	52.7% positive for adenovirus (192 of 364)	Not reported.

© 2022 Alexander EC et al. *JAMA Network Open*.

**Antala et al. (2022)[11]**	United States	n=4	Not stated	4 months – 16 years	2 African American, 2 Caucasian	Children with acute SARS-CoV-2 infection who had severe acute hepatitis as the primary manifestation of COVID-19	Two supportive care; one received eculizumab; one received methylprednisolone	100% survival	None transplanted	All four patients were PCR positive	One patient antibody negative acutely, one IgG positive 6 months later, one IgG positive acutely.	Two of four patient specified tested for adenovirus, both negative.	Not reported.
**Ratho et al. (2022)[12] (Preprint)**	India	n=37 COVID-associated hepatitis in children (CAH-C), n=10 Multisystem Inflammatory Syndrome in children (MIS-C)-associated hepatitis	April 2021-July 2021	Majority aged 2-6 years	Not reported.	MIS-C-associated hepatitis defined as standard definition plus hepatitis. CAH-C was those with laboratory evidence of recent COVID-19 and "sudden onset of hepatitis, elevated transaminases, non-obstructive jaundice, lacking marked inflammatory responses and without evidence of (a) other known causes of acute hepatitis or previous underlying liver disease (b) multi-system involvement".	CAH-C cases were given supportive treatment. MIS-C hepatitis were all treated with steroids, antivirals when needed, and one child received IVIg.	100% survival for CAH-C, 70% survival/ 30% mortality for MIS-C hepatitis.	Not reported.	100% of CAH-C had history of COVID-19 PCR positivity, but 35/37 were PCR negative on admission; 70% of MIS-C hepatitis were PCR positive within 2-3 weeks.	100% of CAH-C (37/37) were antibody positive, 60% (3/5) of MIS-C tested subjects were IgG positive.	17.6% (3/17) of CAH-C were adenovirus PCR positive, 0% (0/3) of MIS-C hepatitis were PCR positive.	Not reported.

© 2022 Alexander EC et al. *JAMA Network Open*.

**van Beek et al. (2022)[13]**	Survey of hospitals in 17 European and 7 non-European countries	n=111 probable cases, n=49 possible cases,	1 January to 25 April 2022	16 years and under.	Not reported.	Acute hepatitis of unknown aetiology	Not reported.	Not reported.	Not reported.	Not reported.	Not reported.	91/126 cases (72%) tested positive for adenovirus from the UK.	Not reported.
**Israel Ministry of Health[14] / Haaretz[15]**	Israel	n=12	1 January to 19th April 2022	Not reported.	Not reported.	Acute hepatitis with no clear background	Children who recovered were reportedly treated with steroids.	Not reported.	Two (16.7%) of 12 children developed liver failure and were transplanted.	Not reported.	Not reported specifically. 11 of 12 children were infected with COVID in the past year.	Not reported.	Not reported.
**Kambhampati et al. (2022)[16]**	United States	Median of 22 and 12 hospitalisations per month in children aged 0-4 and 5-11, respectively with hepatitis of unknown aetiology compared to	October 2021-March 2022	0-11 years	Not reported.	Hepatitis of unspecified etiology using ICD-10-CM codes	Not reported.	Not reported.	Five liver transplant per month during study period compared to four pre-pandemic.	Not reported.	Not reported.	The percentage of specimens positive for adenovirus 40/41 for children 0-9 were the same as pre-pandemic levels.	Not reported.

© 2022 Alexander EC et al. *JAMA Network Open*.

		19.5 and 10.5 in same months pre-pandemic											
**Psaros Einberg and Fischler (2022)[17]**	Sweden	n=9	1 October 2021 – 30 April 2022	Age 0-11, seven below age 5	Not reported.	Acute hepatitis of unknown origin. Patients below 16 without previous liver disease and signs of acute hepatitis. Viral and non-infectious causes excluded.	Not reported.	Not reported.	One of nine patients was transplanted (of a total of 3 who developed acute liver failure)	One patient had active COVID-19 infection.	Not reported.	Two patients were adenovirus PCR positive; none via plasma samples (of n=4 tested). One out of four children tested was positive for adeno-associated virus AAV-2 on metagenomic analysis.	Four patients had biopsies, showing unspecific acute hepatitis. One liver biopsy sample underwent metagenomic analysis without a positive result.

© 2022 Alexander EC et al. *JAMA Network Open*.

**Wollants et al. (2022)[18] (Preprint)**	Belgium	n=9	October 2021 – March 2022	0-15, most commonly 0-4	Not reported.	Children presenting with increased liver enzymes where acute viral hepatitis was the most likely differential diagnosis	Not reported.	100% survival	None transplanted.	One developed SARS-CoV-2 infection 4 days prior to developing hepatitis. Six other patients were negative on PCR, two others not tested.	Not reported.	Six patients tested, all six were negative, from PCR on blood/faeces.	Not reported.
**Lexmond et al. (2022)** [19]	The Netherlands	n=5	March – May 2022	11 months – 8 years	Not reported.	Paediatric Acute Liver Failure of unknown aetiology	Two of five treated with inotropes and kidney replacement therapy.	All five (100%) survived.	Four out of five (80%) were transplanted.	One positive acutely (out of five).	Three out of five were IgG positive (past COVID-19 infection).	Four out of five positive for adenovirus.	Necrosis with ductular reactivity. No signs of chronic liver disease.
**Di Dato et al. (2022)[20]**	Italy	n=34	January – May 2022	Median age 51.5 months, range 1-171	Not reported.	WHO definition of probable cases	Not reported.	All survived.	One patient was transplanted.	Four of 34 (11.8%) were positive.	38.2% had a history of COVID.	6 of 26 were positive for adenovirus (23.1%)	Not reported.

© 2022 Alexander EC et al. *JAMA Network Open*.

**Kelgeri et al. (2022)** [21]	Birmingham, UK	n=44	January 1 – April 11 2022	Median 4 years (range 1-7)	All children were white for whom records were available (80%)	All children 10 years or under who had acute hepatitis consistent with the UKHSA definition	Patients with PALF treated as per standard protocol. Cidofovir was given post-transplant if adenovirus viraemia was >500 copies/ml (for 4/5 children). No children received glucocorticoids or immunomodulators.	100% survived	Overall 6/44 (13.6%) were transplanted.	11/39 (28%) positive on molecular test	5/13 (38%) positive on serological tests	25/27 (93%) were positive for adenovirus on whole blood testing.	Nine children had liver histological analyses (six explants, three biopsies). Patients with PALF who were transplanted showed submassive necrosis with sheets of macrophages replacing the parenchyma. None had viral inclusions and immunohistochemical tests were negative for adenovirus. Three of six explants were tested for adenovirus PCR assays targeting the hexogene and were positive.
**Romaní Vidal et al. (2022)**[22]	20 countries in WHO European region	n=427	1 October 2021-16 June 2022	Mean age 4 years, median 3 years	Not reported.	WHO definition of probable cases	Not reported.	Not reported.	Overall 18 (8.7%) were transplanted.	10.4% of tested subjects were positive for SARS-CoV-2 PCR	66.0% of tested subjects had positive SARS-CoV-2 serology	53.5% of tested subjects were positive for adenovirus	Not reported.
**Ho et al. (2022)**[23] **(Preprint)**	Scotland	n=9 cases, n=58 controls	14 March – 4 April 2022	Cases median 3.9 years	All white ethnicity.	Public Health Scotland definition	Two cases with significant liver dysfunction were given steroids.	100% survived	None were transplanted	2 of 9 were positive for SARS CoV-2 on nucleic acid testing.	6 of 9 children showed serological evidence of prior	All nine had adeno-associated virus 2 (AAV-2) detected in plasma.	AAV-2 was also detected in four of four liver biopsies and adenovirus was detected in 3 of 4 liver biopsy samples.

© 2022 Alexander EC et al. *JAMA Network Open*.

											SARS-CoV-2 exposure.	Adenovirus was detected in 6/9 plasma samples. Higher read counts of HAdV ($p=0.0545$) and AAV2 ($p<0.001$) and HHV6B ($p=0.0059$) were detected in case than control samples.	
**Morfopoulou et al. (2022)[24] (Preprint)**	UK	n=28 cases, n=136 controls	From January 2022	Cases majority <5, up to 10 years	Five transplanted cases were all Caucasian	Aged <10 years with non-A-E hepatitis	Not reported.	Not reported.	Five of 28 cases were transplanted.	Three of five transplanted cases were PCR positive for SARS-CoV-2.	One of five transplanted cases had positive serology. Seven of eight non-transplanted cases had positive serology.	AAV-2 was detected at high levels in blood via PCR from 10/11 non-transplanted cases. Low levels of adenovirus were found in 15/17 blood samples from non transplanted cases. Levels of AAV-2 were significantly higher in cases than controls (Kruskal-Wallis/Wilcoxon test).	AAV-2 was detected in high levels via PCR in explanted livers, and adenovirus in low levels for 5/5 cases. However, no viral particles were identified. No evidence of SARS-CoV-2 infection in liver. Adenovirus was not detected in any control samples and AAV-2 was positive in one.

© 2022 Alexander EC et al. *JAMA Network Open*.

**Cates et al. (2022)[25]**	United States	n=296	October 1 2021 – June 15 2021	Median age 2 years 2 months (range 1 months – 9 years 8 months)	Hispanic/ Latino 37.8%, White, non-Hispanic 32.4%, Black, non-Hispanic 9.8%	Patients aged <10 years with hepatitis of unknown aetiology as per CDC	Not reported.	11 patients (3.7%) died.	18 patients (6.1%) received a liver transplant.	10 of 98 tested cases were positive acutely for SARS-CoV-2 (10.2%)	32 cases (26.0%) had a history of SARS-CoV-2 infection	48 of 97 were positive for adenovirus from any specimen time (49.5%)	25 of 36 patients with liver analysis showed evidence of active/acute hepatitis, none with viral inclusions.

Note: The same patient may appear across multiple studies; included studies/reports are those published in the English language; not all reports have been peer-reviewed; in some cases accepted version only was available

© 2022 Alexander EC et al. *JAMA Network Open*.

eReferences

1. Deep A, Grammatikopoulos T, Heaton N, Verma A, Dhawan A. Outbreak of hepatitis in children: clinical course of children with acute liver failure admitted to the intensive care unit. *Intensive Care Medicine.* 2022.
2. UK Health Security Agency. Investigation into acute hepatitis of unknown aetiology in children in England: Technical briefing 4. https://assets.publishing.service.gov.uk/government/uploads/system/uploads/attachment_data/file/1094573/acute-hepatitis-technical-briefing-4.pdf. Published 2022. Accessed 30th August 2022.
3. Cooper S, Tobar A, Konen O, et al. Long COVID-19 Liver Manifestation in Children. *Journal of Pediatric Gastroenterology and Nutrition.* 2022.
4. de Kleine RH, Lexmond WS, Buescher G, et al. Severe acute hepatitis and acute liver failure of unknown origin in children: a questionnaire-based study within 34 paediatric liver centres in 22 European countries and Israel, April 2022. *Euro Surveill.* 2022;27(19):2200369.
5. Marsh K, Tayler R, Pollock L, et al. Investigation into cases of hepatitis of unknown aetiology among young children, Scotland, 1 January 2022 to 12 April 2022. *Eurosurveillance.* 2022;27(15):2200318.
6. CDC Health Alert Network. Updated Recommendations for Adenovirus Testing and Reporting of Children with Acute Hepatitis of Unknown Etiology. https://emergency.cdc.gov/han/2022/han00465.asp. Published 2022. Accessed 12 June 2022.
7. Baker JM, Buchfellner M, Britt W, et al. Acute Hepatitis and Adenovirus Infection Among Children - Alabama, October 2021-February 2022. *MMWR Morb Mortal Wkly Rep.* 2022;71(18):638-640.
8. Baker JM, Buchfellner M, Britt W, et al. Acute hepatitis and adenovirus infection among children—Alabama, October 2021–February 2022. *American Journal of Transplantation.* 2022;22(7):1919-1921.
9. Gutierrez Sanchez LH, Shiau H, Baker JM, et al. A Case Series of Children with Acute Hepatitis and Human Adenovirus Infection. *New England Journal of Medicine.* 2022.
10. Joint ECDC-WHO Regional Office for Europe. Joint ECDC-WHO Regional Office for Europe Hepatitis of Unknown Origin in Children Surveillance Bulletin. https://cdn.ecdc.europa.eu/novhep-surveillance/. Published 2022. Updated 1 July 2022. Accessed 17 July, 2022.
11. Antala S, Diamond T, Kociolek LK, Shah AA, Chapin CA. Severe Hepatitis in Pediatric Coronavirus Disease 2019. *Journal of pediatric gastroenterology and nutrition.* 2022;74(5):631-635.
12. Ratho RK, Asati AA, Mishra N, Jain A, Rawat SK. COVID-19 Associated Hepatitis in Children (CAH-C) during the second wave of SARS-CoV-2 infections in Central India: Is it a complication or transient phenomenon. *medRxiv.* 2022:2021.2007.2023.21260716.
13. van Beek J, Fraaij PL, Giaquinto C, et al. Case numbers of acute hepatitis of unknown aetiology among children in 24 countries up to 18 April 2022 compared to the previous 5 years. *Eurosurveillance.* 2022;27(19):2200370.
14. Ministry of Health. The Ministry of Health Informed of 12 Child Cases with Acute Hepatitis with no Clear Background. https://www.gov.il/en/departments/news/19042022-01. Published 2022. Accessed 14th June 2022.
15. Haaretz. Israel Examining 12 Cases of Kids' Hepatitis After WHO Warning. https://www.haaretz.com/israel-news/2022-04-21/ty-article/israel-examining-12-cases-of-kids-hepatitis-after-who-warning/00000180-6567-d5ca-a986-7f6f8a990000. Published 2022. Accessed 14th June 2022.
16. Kambhampati AK, Burke R, Dietz S, et al. Trends in Acute Hepatitis of Unspecified Etiology and Adenovirus Stool Testing Results in Children — United States, 2017–2022. *MMWR Morb Mortal Wkly Rep.* 2022;71.
17. Psaros Einberg A, Fischler B. Acute hepatitis of unknown origin – what we know so far. *Acta Paediatrica.* 2022;n/a(n/a).

© 2022 Alexander EC et al. *JAMA Network Open*.

18. Wollants E, Keyaerts E, Cuypers L, et al. Environmental circulation of adenovirus 40/41 and SARS-CoV-2 in the context of the emergence of acute hepatitis of unknown origin. *medRxiv.* 2022:2022.2006.2008.22276091.
19. Lexmond WS, de Meijer VE, Scheenstra R, et al. Indeterminate pediatric acute liver failure: Clinical characteristics of a temporal cluster of five children in the Netherlands in the spring of 2022. *United European Gastroenterology Journal.* 2022;n/a(n/a).
20. Di Dato F, Di Giorgio A, Mandato C, et al. Italian children seem to be spared from the mysterious severe acute hepatitis outbreak: a report by SIGENP Acute Hepatitis Group. *Journal of Hepatology.* 2022.
21. Kelgeri C, Couper M, Gupte GL, et al. Clinical Spectrum of Children with Acute Hepatitis of Unknown Cause. *New England Journal of Medicine.* 2022.
22. Romaní Vidal A, Vaughan A, Innocenti F, et al. Hepatitis of unknown aetiology in children – epidemiological overview of cases reported in Europe, 1 January to 16 June 2022. *Eurosurveillance.* 2022;27(31):2200483.
23. Ho A, Orton R, Tayler R, et al. Adeno-associated virus 2 infection in children with non-A-E hepatitis. *medRxiv.* 2022:2022.2007.2019.22277425.
24. Morfopoulou S, Buddle S, Torres Montaguth OE, et al. GENOMIC INVESTIGATIONS OF ACUTE HEPATITIS OF UNKNOWN AETIOLOGY IN CHILDREN. *medRxiv.* 2022:2022.2007.2028.22277963.
25. Cates J, Baker JM, Almendares O, et al. Interim analysis of acute hepatitis of unknown etiology in children aged <10 years - United States, October 2021-June 2022. *MMWR Morb Mortal Wkly Rep.* 2022;71(26):852-858.

© 2022 Alexander EC et al. *JAMA Network Open*.
